# Assessment of oral emergency services during COVID-19: a retrospective study of 14,885 cases in Shanghai

**DOI:** 10.1186/s12903-023-03563-1

**Published:** 2023-11-06

**Authors:** Jian Wang, Jun-Jun Zhao, Zhao-Wei Tai, Xue-Chun Wang, Jiang Tao, Qian Liao

**Affiliations:** 1 Department of General Dentistry, Shanghai Ninth People’s Hospital, School of Medicine, College of Stomatology, Shanghai Jiao Tong University, National Center for Stomatology, National Clinical Research Center for Oral Diseases, Shanghai Key Laboratory of Stomatology, Shanghai Research Institute of Stomatology, Research Unit of Oral and Maxillofacial Regenerative Medicine, Chinese Academy of Medical Sciences, Shanghai, 200011 China; 2 Department of Stomatology, Shanghai General Hospital, Shanghai Jiao Tong University School of Medicine, Shanghai, 200011 China

**Keywords:** COVID-19, Oral, Emergency

## Abstract

**Background:**

To assess the impact of coronavirus disease-2019 (COVID-19) in its outbreak stage (Spring Festival in 2020) on oral emergency services.

**Methods:**

Oral emergency cases in Shanghai Ninth People’s Hospital, Shanghai Jiao Tong University School of Medicine, during the Spring Festival after the outbreak of the COVID-19 epidemic in 2020 were collected and compared with those in 2018 and 2019. Electronic medical records including the visited department, age, sex, time, date, region, and diagnosis were collected and analyzed. The results were statistically analyzed using Pearson’s Chi-square test and one-way analysis of variance (ANOVA).

**Results:**

Compared with that in 2018 and 2019, the total number of patients decreased during the Spring Festival in 2020 (p < 0.001), but the proportions of patients visiting Oral Surgery and Oral, Head, and Neck Oncology Emergency departments increased. The average age of patients increased, and the number of night visits decreased. Toothache diseases involving endodontic and periodontal diseases increased, while the proportion of maxillofacial trauma decreased. The wasn’t a linear association between diagnosis or genders (P > 0.001) across years. However, a linear-by-linear association between age groups and years, visited departments and years were observed (P < 0.001).

**Conclusions:**

The study revealed that the transmission of COVID-19 affected the patient population and structure of disease types and oral services in 2020 during the Spring Festival, compared with those in the previous two years. The visits to oral emergency departments and the proportions of patients who were children and adolescents reduced; meanwhile, the percentage of the elderly people increased during the outbreak of COVID-19. The clear trend of age groups and visiting divisions could be used as a marker to reflect the severity of the COVID-19 pandemic. These results may serve as a reference for dental practitioners involved in oral emergency services and to allocate the limited emergency health resources.

## Background

Dental practitioners are vulnerable to pathogenic microorganisms originating from the oral cavity and respiratory tract during dental visits and regular procedures. The extremely high risk of exposure to blood and saliva and direct contact with patients creates dangerous circumstances for dental professionals, especially when resolving oral hemorrhages and maxillofacial injuries in the oral emergency department [1–4].

Coronavirus disease 2019 (COVID-19), first reported in China in December 2019, has become an emerging challenge and caused a pandemic [5, 6]. As a pneumonia-related disease, infected individuals usually present with fever, cough, and fatigue on abnormal chest computed tomography (CT) images after an incubation period of 2–14 days. Others may also experience sputum production, loss of taste, headache, hemoptysis, and diarrhea [6–8]. Elderly individuals, especially those with systemic diseases or who are immunocompromised, are vulnerable to COVID-19 [9, 10]. Dental professionals, especially doctors in oral emergency departments, have a high risk of exposure to COVID-19 and remain essential workers during the pandemic.

The Chinese lunar Spring Festival, which is the most important holiday for the Chinese people, has special significance for family reunions in China’s social-cultural context. Many migrant residents return to their hometowns to reunite with their families, which is commonly observed in China’s megacities before festivals. After the festival, they return to their cities and continue to study or work. This phenomenon is known as “Spring Travel” or “Chunyun” in China. However, 2020 began with the COVID-19 lockdown that overlapped with the traditional Chinese Spring Festival [11–13]. The COVID-19 pandemic changed human behavior in many ways during the Spring Festival in 2020 [4, 14]. To have a comprehensive understanding of the impact of COVID-19 on oral emergency services and to investigate oral emergency patients’ relevant clinical characteristics, a retrospective study at Shanghai Ninth People’s Hospital during the Spring Festival from 2018 to 2020 was conducted; it aimed to provide crucial suggestions for the diagnosis, treatment, and further scientific research of oral emergency.

## Methods

### Data source

Patients’ electronic medical histories were stored and protected in the electronic medical history database of Shanghai Ninth People’s Hospital. This retrospective cross-sectional study was approved and authorized by the Independent Ethics Committee of the Shanghai Ninth People’s Hospital, Shanghai Jiao Tong University School of Medicine. The project was conducted at Shanghai Ninth People’s Hospital, the largest oral emergency center in Shanghai, which provides 24-h access to immediate oral emergency services.

### Sample selection

The study included patients who visited the oral emergency department of Shanghai Ninth People’s Hospital during the Spring Festival (from Chinese New Year’s Eve to the subsequent 26 days [27 days in total] in 2018, 2019, and 2020).

The specific time information was as follows:

- February 15^th^ – March 14^th^ in 2018.
- February 4^th^ – March 3^rd^ in 2019.
- January 24^th^ – February 20^th^ in 2020.

### Inclusion and exclusion criteria

Patients with complete electronic medical records including the department visited, age, sex, region, time of visit, and diagnosis were included. Patients with incomplete electronic medical records or those missing one or more pieces of information mentioned above were excluded.

### Variables

Patient information, including the department visited, age, sex, region, time of visit, and diagnosis, were retrieved from a local electronic medical history database. The study was conducted in four divisions responsible for treating oral emergency patients (General Dentistry; Oral Surgery; Craniomaxillofacial Surgery; Oral, Head, and Neck Oncology). Patients were divided into three subgroups based on age: minors (≤ 17 years), adults (18–59 years) and the elderly (≥ 60 years). The regions were classified into three parts: Shanghai, six provinces in Eastern China, and other regions. Visiting time was analyzed at intervals of 2 h, and diagnosis was classified according to the International Classification of Diseases (ICD-10).

In the current study, patients with oral emergency diseases were divided into 10 groups labelled A to J. Detailed information on disease types were provided as follows:

(A) various maxillofacial injuries, soft tissue injuries, and bone fractures.
(B) maxillofacial infections, including swelling induced by inflammation of maxillofacial tissues, abscess, and multi-space infections.
(C) acute toothache, including pulpitis and periapical or periodontal pain.
(D) pericoronitis, mainly caused by an impacted third molar.
(E)  acute tooth injuries, including tooth fracture, dislocation, and concussion.
(F)  oral bleeding caused by various reasons.
(G) temporomandibular joint emergencies.
(H) emergencies caused by oral masses mainly visited the Oral, Head, and Neck Oncology Emergency department.
(I)  acute oral mucosal diseases, parotitis, and erysipelas.
(J)  non-urgent diseases, such as caries, residual crowns, residual roots, and dentition defects, who could receive outpatient treatment.

### Statistics

SPSS Statistics (version 25.0, IBM Corp., USA) software was used for statistical analysis. Continuous variables are represented by x ± s, and categorical variables are statistically described by number of examples and percentages. The difference between patient characteristics groups in different years was analyzed by Pearson’s Chi-square test and one-way ANOVA, and stratified by age, with p ≤ 0.05 set as statistically significant.

## Results

Of the 14,885 patients in this study, 7963 male and 6922 female patients were included, with a male-to-female ratio of 1.15:1. The average age of the visitors was 40.3 ± 22.7 years, with the youngest individual being aged 1 year and the oldest being aged 98 years.

During the Spring Festival in 2018, 2019, and 2020, the number of oral emergency visits was 5520, 5535, and 3830, respectively; the number of patients in 2020 was significantly lower than that in the former two years (p < 0.001). No significant difference was identified in the sex distribution of oral emergency patients across the three years (p = 0.209). Regarding emergency departments, the departments visited across the three years were not uniform, and the difference was statistically significant (p < 0.001). The proportion of emergency patients in the departments of Oral Surgery and Oral, Head, and Neck Oncology in 2020 was higher than that in the previous two years. The average age of patients in 2020 was also higher than that of the previous two years (p < 0.001), and the proportion of elderly people aged ≥ 60 years was 29.6%, significantly higher than that of the previous two years (2018, 23.8%; 2019, 23.7%; p < 0.001) (Table 1).

**Table 1:** Basic demographic characteristics of oral emergency patients from 2018 to 2020 [n (%)]

Year		2018	2019	2020	c^2^/F value	P value
Age		39.9 ± 22.4	39.2 ± 22.8	42.6 ± 22.7	26.522	<0.001^b^
Age Groups	Minors (< 18years)	1103(20.0)	1135(20.5)	647(16.9)	58.572	<0.001^a^
	Adults (18–59 years)	3106(56.3)	3086(55.8)	2050(53.5)		
	The elderly ( > = 60 years)	1311(23.8)	1314(23.7)	1133(29.6)		
Gender	Male	2930(53.1)	2937(53.1)	2096(54.7)	3.132	0.209^a^
	Female	2590(46.9)	2598(46.9)	1734(45.3)		
	Ratio (Male: Female)	1.13:1	1.13:1	1.21:1		
Divisions	Dental Emergency	3705(67.1)	3927(70.9)	2511(65.6)	226.07	<0.001^a^
	Cranio-Maxillofacial Surgery Emergency	1354(24.5)	1228(22.2)	741(19.3)		
	Oral Surgery Emergency	428(7.8)	359(6.5)	512(13.4)		
	Oral, Head and Neck Oncology Emergency	33(0.6)	21(0.4)	66(1.7)		
Regions	Shanghai	2843(67.5)	2839(65.0)	2227(70.4)	35.353	<0.001^a^
	6 eastern cities	865(20.6)	955(21.9)	530(16.8)		
	others	501(11.9)	571(13.1)	405(12.8)		
Total		5520(100.0)	5535(100.0)	3830(100.0)		

a: χ^2^ test b: ANOVA

**Fig. 1:**
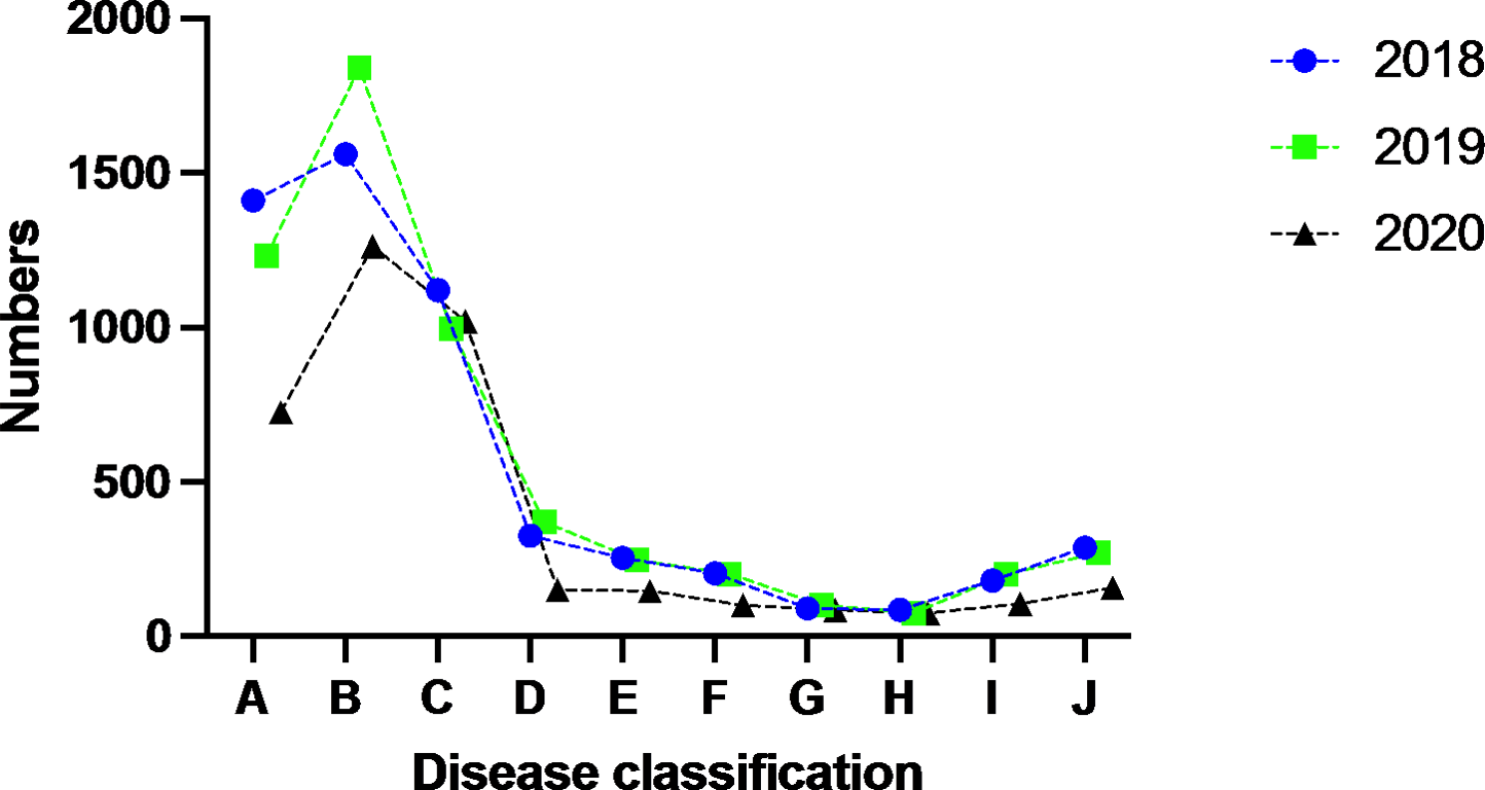
Disease types’ distribution of oral emergency visits from 2018 to 2019

Regarding time distribution, intervals of 2 h were used for data description. The patients’ visit times were not completely the same across the three years, and the difference was statistically significant (χ^2^=381.153, p < 0.001). After comparison in pairs, the time distributions of oral emergency visits in 2018 and 2019 were similar (χ^2^=11.116, p = 1.000), and the difference was statistically significant in the temporal distributions of oral emergency visits between 2020 and both 2018 and 2019 (2019 vs. 2020: χ^2^=317.702, p < 0.001; 2018 vs. 2020: χ^2^=247.706, p < 0.001). The total number of oral emergency department visits in 2019 was essentially the same as that in 2018 (increase of 0.27%), while that in 2020 decreased by 30.80%, compared with 2019 (Fig. 1).

Regarding time distribution, the volume of oral emergency visits decreased significantly from 16:00 in 2020 to the highest peak at 8:00–10:00 a.m. the next morning, while the overall volume of oral emergencies between 14:00–22:00 in 2018 and 2019 remained at a high level (Fig. 1).

**Fig. 2:**
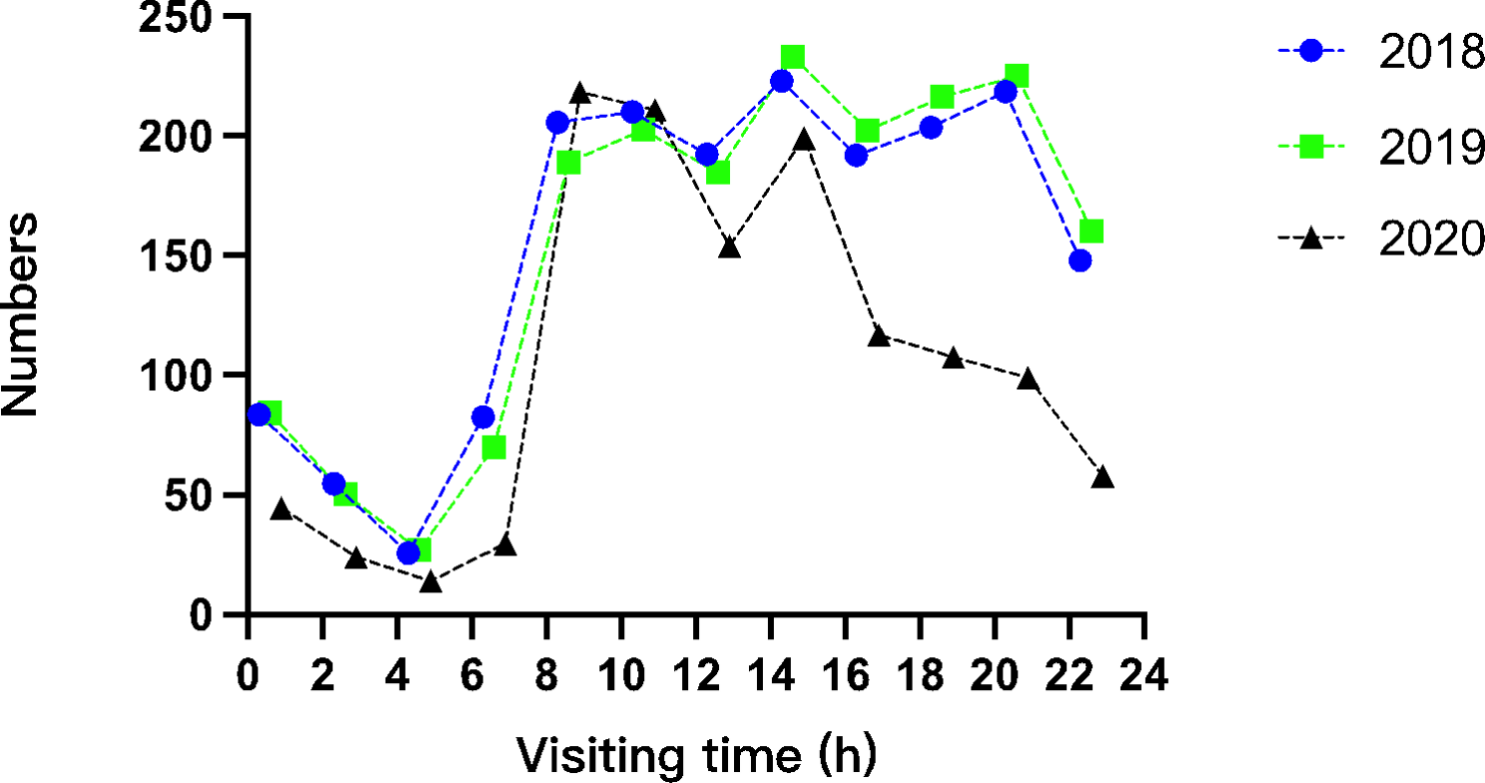
Time distribution of oral emergency visits from 2018 to 2019

The main causes of oral emergency department visits were maxillofacial infection (4622, 31.1%) and maxillofacial trauma (4045, 27.2%), followed by apical periodontal (1122, 7.5%), endodontic (1093, 7.5%), and periodontal (818; 5.5%) diseases. Significant differences were identified in the distribution of etiology among patients (χ^2^=352.038, p < 0.001); compared with the previous two years, the proportions of apical periodontal disease and endodontic disease increased in 2020, while the proportion of maxillofacial trauma decreased significantly (Table 2; Fig. 2).

**Table 2:** Information of disease types of oral emergency

Types	2018	2019	2020	Total
A	1410(25.54%)	1231(22.24%)	725(18.93%)	3366(22.61%)
B	1561(28.28%)	1840(33.24%)	1262(32.95%)	4663(31.33%)
C	1121(20.31%)	995(17.98%)	1021(26.66%)	3137(21.07%)
D	326(5.91%)	370(6.68%)	151(3.94%)	847(5.69%)
E	254(4.60%)	248(4.48%)	147(3.84%)	649(4.36%)
F	204(3.70%)	203(3.67%)	100(2.61%)	507(3.41%)
G	91(1.65%)	102(1.84%)	86(1.84%)	279(1.87%)
H	86(1.56%)	75(1.36%)	75(1.96%)	236(1.59%)
I	180(3.26%)	200(3.61%)	106(2.77%)	486(3.27%)
J	287(5.30%)	271(4.90%)	157(4.10%)	715(4.80%)
Total	5520(100%)	5535(100%)	3830(100%)	14,885(100%)

In the subgroup analysis by age, a significant difference was observed in the distribution of etiologies between the age subgroups (p < 0.001). The causes of oral consultations varied among patients of different ages, with maxillofacial trauma accounting for the highest proportion in the minor group, followed by maxillofacial infections. Maxillofacial infections accounted for the highest proportion of diseases in adult and elderly groups, followed by maxillofacial trauma. Pericoronitis has the highest incidence in adults.

**Table 3:** Chi-square trend analysis of oral emergency

Factors	Value	P
Disease types	0.585	0.445
Genders	2.156	0.142
Age groups	35.064	< 0.001
Visited divisions	31.524	< 0.001

Table 3 showed the Chi-square trend test about disease types, genders, age groups, and departments visited over years. There was no linear association between disease types or genders (p > 0.001) across years. However, a linear-by-linear association between age groups and years, departments visited and years were observed (p < 0.001).

## Discussion

The Spring Festival, one of the most important traditional Chinese holidays, is a large event celebrated throughout the country. Under these circumstances, when facing oral complications, most patients had to go to oral emergency centers to obtain diagnoses and treatment, as private dental departments and clinics were suspended during holiday [15–17], and COVID-19 policies following its outbreak in 2020 included the limitation of outdoor activities. In Shanghai, patients requiring urgent care can only seek oral emergency services at the Shanghai Ninth People’s Hospital, Shanghai Jiao Tong University School of Medicine.

As the largest public oral clinic center in Shanghai, Shanghai Ninth People’s Hospital has divided the emergency department into four divisions since 2017 [18]: Dental Emergency; Oral Surgery Emergency; Craniomaxillofacial Surgery Emergency; and Oral, Head, and Neck Oncology Emergency. Dental Emergency mainly involves diseases such as toothaches, tooth injuries, and oral infections, whereas Oral Surgery Emergency treats oral multi-space infections. Craniomaxillofacial Surgery Emergency treats various maxillofacial injuries, and Oral, Head, and Neck Oncology Emergency is responsible for cases involving tumors. The four divisions play constructive roles every day to treat citizens’ urgent oral diseases and to better serve the people of Shanghai and surrounding areas. This study was conceived and designed to gain a better understanding of the impact of the environment, both during and outside the COVID-19 pandemic, on oral emergency services during the Chinese Spring Festival.

Regarding the numbers of oral emergency visits, a similar study conducted by Bai J et al. in Beijing reported a slight reduced number of oral emergency visits (decrease of 7.5%) during 2020’s Spring Festival, compared with that during the same time in 2019 [16]. Moreover, we observed a 30.8% decrease in the number of oral emergency visits during this period. When investigating the reasons behind the results in January and February 2020, more daily reports confirmed that the threat of COVID-19 in Shanghai reminded citizens of a higher risk due to its high transmissibility [13, 19]; therefore, citizens were more reluctant to participate in outdoor activities to avoid the unnecessary danger of encountering COVID-19, especially visiting hospitals. Bai et al. calculated data for the statutory holiday period of the Spring Festival in 2019 and 2020. In 2020, the government extended its original 7-day-holiday to 10 days owing to the COVID-19 epidemic. The additional 3 days of visiting patients during the 2020 Spring Festival may have narrowed the actual difference. Another study documented that the total number of cases decreased by more than 25% [20], which also supports our results. In our study, the male-to-female ratio was 1.13:1 in 2018 and 2019 and 1.21:1 in 2020. Shen et al. conducted a survey among 137,740 cases in Shanghai between 2017 and 2018 [18], with a ratio of 1.13:1. Dong et al. reported a male-to-female ratio of 1.10–1.17:1 in a four-year investigation of the first half (first six months) from 2017 to 2020 [21]. These results are consistent with those of this study.

Regarding visiting times, a significant difference was observed between 2020 and the previous two years (Chi-square test, χ^2^ = 381.153, p < 0.001). In general, the number of oral health emergency patients within one day showed two high peaks (8:00–12:00 and 18:00–22:00) and one low peak (0:00–6:00). During the pandemic, oral emergency visits decreased significantly from 16:00 to a peak at 8:00–10:00 the following day, and night oral emergency visits were significantly affected. After the implementation of a first-level response mechanism to major public health emergencies against COVID-19 on January 24th, 2020 [13], citizens followed the government’s instructions and stayed at home during the day, and most individuals chose to visit the hospital between 8:00 and 10:00. The pandemic has had a potential impact on patient preferences regarding visiting times. Elderly individuals were the least affected compared with minors and adults.

Regarding the four department divisions, as depicted in Table 1, the number of visitors to Oral Surgery and Oral, Head, and Neck Oncology increased, whereas the number of patients undergoing Dental Emergency and Craniomaxillofacial Surgery decreased significantly. As traffic accidents and trauma dropped to low levels during the 2020 pandemic under preventive and control policies, the number of patients with toothaches and various injuries decreased. The increase in the proportion of visits for maxillofacial infections and tumors may be attributed to a mild delay in medical treatment, resulting in the exacerbation of the disease and the development of urgent and life-threatening clinical symptoms, especially in the elderly with underlying diseases. Several studies have reported that the proportion of women requiring dental services was higher during the non-pandemic period than during the pandemic period [22]. Under normal conditions, women have broader concerns about oral health and are inclined to visit dentists more often than men [19, 23]. However, owing to fear of COVID-19, women sought dental emergency services less often during the COVID-19 pandemic. In the current study, we observed a slightly higher ratio of men than women in all three years, which is consistent with the results of Guo et al. [19].

The COVID-19 pandemic has had a direct impact on oral emergency services. A clear negative relation was observed between age groups and years using Chi-square trend tests, as was the visiting department (p < 0.001). A clear trend in age groups and visiting divisions could be used as markers to reflect the severity of the COVID-19 pandemic. For instance, when the pandemic increased, a negative correlation between age groups and visiting departments of oral emergency services was observed. This perspective will help us to gain a better understanding of the long-term influence of the pandemic on oral emergency services.

Although our retrospective study successfully revealed the different characteristics of oral emergency patients, some limitations of this study must be addressed. First, when we reviewed the electronic records, some patients complained of toothache with actual diagnoses of acute pulpitis or acute apical periodontitis, which ultimately led to maxillofacial infections and antibiotic use to relieve local pain. This phenomenon may decrease the incidence of toothache-related diseases and increase the incidence of maxillofacial infections. This could have potentially biased the distribution of diseases. Second, in the current study, we only investigated the impact of COVID-19 during the Spring Festival over three years and differences in medical compliance of emergency patients were not examined, and subsequent outpatient treatment was also unclear. Further studies could provide a more in-depth evaluation of dental treatment completion rates in patients using oral emergency services. Lastly, some potential confounding factors that may have influenced patients’ decisions to use oral emergency services included socioeconomic status and access to oral healthcare during the Spring Festival, which are worthy of further investigation. Although the aforementioned limitations or other factors could have influenced our results, the present study has some notable strengths. First, the study retrospectively compared oral emergency services during the Spring Festival over three years and narrowed the gap between research and medical procedures, providing healthcare systems with a general understanding of what constitutes an oral emergency to guarantee that patients access essential care and to establish practical solutions for determining appropriate care locations. Furthermore, hospitals and clinics should establish plans for unprecedented pandemics that involve echelon reserves of emergency staff in various departments, particularly to strengthen the reserves of medical resources, improve oral public health emergency management capabilities, and reduce the risk of infection between doctors and patients. The current work could be a window providing meaning insights for healthcare leaders to develop actionable and clear strategies when facing up with the potential crisis. Detailed outlines such as setting guidelines for public oral health campaigns during crises, preparing adaptive strategies for oral healthcare during times of societal upheaval, and specific training or preparation modules for oral healthcare professionals to handle future events such as COVID-19.

The Discussion section elucidates the relationship between the pandemic and its impact on oral emergency services. We can draw a further inference that once confronted with unforeseen crises or pandemics, such as COVID-19, in the future, dental practitioners and the oral healthcare industry will have broader implications. A deeper analysis involving the investigation of subsequent dental treatment completion rates and the factors influencing patients’ decision-making processes for seeking emergency dental care will be considered in future studies to better elucidate oral emergency services. More specifically, qualitative studies involving patient behavior or longitudinal investigations into the long-term impact of disrupted dental care during the pandemic could also widen the avenues for future research.

## Conclusions

In conclusion, this study revealed that the transmission of COVID-19 affected the patient population, structure of disease types, and oral services in 2020 during the Spring Festival compared with those in the previous two years. The number of visits to oral emergency departments and the proportion of children and adolescents decreased, whereas the proportion of elderly people increased during the outbreak of COVID-19. A clear trend in age groups and visiting divisions could be used as markers to reflect the severity of the COVID-19 pandemic. These results may serve as a reference for dental practitioners in providing oral emergency services and allocating limited emergency health resources.

## Data Availability

The datasets used and/or analyzed during the current study available from the corresponding author on reasonable request.

## List of Abbreviations

ANOVA: Analysis of variance
COVID-19: Coronavirus disease 2019
CT: Computed tomography
